# National variation in pulmonary metastasectomy for colorectal cancer

**DOI:** 10.1111/codi.15506

**Published:** 2021-01-24

**Authors:** Hayley M. Fenton, Paul J. Finan, Richard Milton, Michael Shackcloth, John C. Taylor, Tom Treasure, Eva J. A. Morris

**Affiliations:** ^1^ Cancer Epidemiology Group Leeds Institute for Data Analytics University of Leeds Leeds UK; ^2^ Department of Thoracic Surgery St James’s University Hospital Leeds UK; ^3^ Department of Thoracic Surgery Liverpool Heart and Chest Hospital Liverpool UK; ^4^ Clinical Operational Research Unit University College London London UK; ^5^ Nuffield Department of Population Health Big Data Institute University of Oxford Oxford UK

**Keywords:** colorectal cancer, pulmonary metastasectomy, pulmonary metastases

## Abstract

**Aim:**

Evidence on patterns of use of pulmonary metastasectomy in colorectal cancer patients is limited. This population‐based study aims to investigate the use of pulmonary metastasectomy in the colorectal cancer population across the English National Health Service (NHS) and quantify the extent of any variations in practice and outcome.

**Methods:**

All adults who underwent a major resection for colorectal cancer in an NHS hospital between 2005 and 2013 were identified in the COloRECTal cancer data Repository (CORECT‐R). All inpatient episodes corresponding to pulmonary metastasectomy, occurring within 3 years of the initial colorectal resection, were identified. Multi‐level logistic regression was used to determine patient and organizational factors associated with the use of pulmonary metastasectomy for colorectal cancer, and Kaplan–Meier and Cox models were used to assess survival following pulmonary metastasectomy.

**Results:**

In all, 173 354 individuals had a major colorectal resection over the study period, with 3434 (2.0%) undergoing pulmonary resection within 3 years. The frequency of pulmonary metastasectomy increased from 1.2% of patients undergoing major colorectal resection in 2005 to 2.3% in 2013. Significant variation was observed across hospital providers in the risk‐adjusted rates of pulmonary metastasectomy (0.0%–6.8% of patients). Overall 5‐year survival following pulmonary resection was 50.8%, with 30‐day and 90‐day mortality of 0.6% and 1.2% respectively.

**Conclusions:**

This study shows significant variation in the rates of pulmonary metastasectomy for colorectal cancer across the English NHS.

**What does this paper add to the literature?**

There is limited population‐based evidence available on patterns of pulmonary metastasectomy for colorectal cancer lung metastases. This is one of the first studies to use large population‐level, linked datasets to investigate practice and outcomes following such surgery across the English National Health Service.

## INTRODUCTION

Colorectal cancer is the fourth most common cancer in the UK, with around 42 000 new cases per year, and the second leading cause of cancer‐related death [1]. Approximately 20% of colorectal cancer patients will have metastases at diagnosis and they will subsequently become apparent in up to 50% of people [2]. Around 5%–15% of patients with cancer of the colon will develop metastases in the lungs during the disease course, and such spread is proportionally more common in rectal cancer patients, because of the difference in venous drainage between colon and rectum, with reported figures of between 11% and 19% [2, 3]. Lung only or, probably more correctly, lung‐first metastases are uncommon in colorectal cancer, however, with around 80%–85% of patients also having metastases in other sites, most frequently the liver [2].

There are several important recent influences on surgical practice within the National Health Service (NHS) with respect to colorectal lung metastases. First, the Association of Coloproctology of Great Britain and Northern Ireland (ACPGBI), in its Improving Management of Patients with Advanced Colorectal Tumours (IMPACT) initiative in 2017, has promoted an active policy of monitoring, detecting and team discussions to offer further treatment [4]. A Delphi project run by the ACPGBI ranked management of lung metastases high in its research priorities [5]. Second, the National Institute for Health and Clinical Excellence (NICE), which provides guidance for the NHS, published guidance on the management of metastatic disease in colorectal cancer in January 2020. They found no randomized trials and based their guidance on a ‘very low quality’ follow‐up analysis [6] but still recommended that lung metastasectomy should be ‘considered’ [7]. Finally, the Society of Thoracic Surgeons Consensus Document on Pulmonary Metastasectomy in 2019 [8] again, despite acknowledging the lack of randomized evidence, gave as their first recommendation that pulmonary metastasectomy should be ‘considered’ and ‘carefully individualized’ [8].

‘Pulmonary metastasectomy in colorectal cancer’, published in 2020 [9, 10], was the first randomized trial on this topic. Patients were randomized to lung metastasectomy (*N* = 46) or control (*N* = 47) and 87% were followed for more than 5 years or until death. There was no significant difference comparing metastasectomy to controls; the hazard ratio (HR) for death was 0.93 (95% CI 0.56, 1.56). This puts into question the recommendations for practice from ACPGBI, Society of Thoracic Surgeons and NICE.

It had been estimated that 15%–25% of those with pulmonary metastases were being considered for local treatments [11]. Traditionally surgery has been the main local treatment, but more recently image guided thermal ablation (IGTA), including radiofrequency, microwave or cryoablation, and stereotactic radiotherapy (variously abbreviated as SABR or SBRT) have been increasingly used as alternatives. Each has been tested in a randomized controlled trial [12, 13] but, unfortunately, both had major imbalances across trial arms so, again, it is not clear what constitutes gold standard care.

Further studies with an emphasis on understanding the extent and characteristics of patients with metastases are required. Arguably more information is required about the disease in its totality rather than outcomes amongst patients carefully selected for local treatments. Most existing studies on pulmonary metastasectomy are single‐centre surgical case series [8] with very few population‐based cohorts or registry studies [14, 15, 16, 17]. When the larger series within these studies are considered, the incidence of pulmonary metastasectomy is estimated to be only around 2.5% [14, 16] of all colorectal cancer patients and 3.5%–5% [14, 17] of those with metastases at diagnosis. As such, pulmonary metastasectomy is clearly used in a highly selectively manner.

This population‐based study therefore aimed to investigate the use of pulmonary metastasectomy in the colorectal cancer population across the English NHS and to quantify the extent of any variations in practice and outcome.

## METHODS

All adults diagnosed with a first primary colorectal cancer (ICD‐10 codes C18–C20), and who had undergone a major resection for their disease in an NHS hospital with a multidisciplinary team (MDT) between 1 January 2005 and 31 December 2013 (to allow 3 years of follow‐up until censoring at December 2016), were identified in the COloRECTal cancer data Repository (CORECT‐R). This resource contains numerous linked population‐level datasets relevant to colorectal cancer and, for this study, information was derived from a linked National Cancer Registration and Analysis Service (NCRAS) and Hospital Episode Statistics (HES) dataset. Information on date of diagnosis, age, sex, site and stage of tumour and deprivation (measured via the income domain of the Index of Multiple Deprivation [IMD] 2010), were extracted from the cancer registry dataset. Where patients had multiple tumours recorded simultaneously, the tumour with the highest stage was selected. Any remaining duplicate patient records were cleaned to select the most relevant tumour for the type of major resection carried out. Information on the type and date of the first major resection surgery following diagnosis was extracted from the HES component of CORECT‐R. Major primary resection for colorectal cancer and lung resection were identified by the appropriate OPCS 4.8 codes (Appendix Table S1). The lung resection codes were presumed to correspond to pulmonary metastasectomy. It is possible that some of these procedures may have been carried out for primary lung cancer or other diagnoses, due to the absence of additional information. Primary tumours of the caecum, appendix, ascending colon, hepatic flexure and transverse colon (ICD‐10 codes C18.0–C18.4) were assigned as right‐sided colon tumours, whilst tumours in the splenic flexure, descending colon, sigmoid colon and rectosigmoid (ICD‐10 C18.5–C19) were assigned as left‐sided colon tumours. Rectal tumours were assigned using ICD‐10 C20. Where more than one OPCS procedure code appeared on the same day for the lung resection, the most extensive operation was selected.

The Charlson Comorbidity Index (CCI) [18] was derived for each patient, taking into account diagnoses (excluding cancer) from any hospital admissions in the year preceding diagnosis of colorectal cancer. The cancer component of the CCI was derived from the cancer registry information in CORECT‐R and added to that obtained from HES data. The CCI was categorized as 0, 1, 2 and ≥3 with higher scores indicating greater degree of comorbidity.

Data from the Organizational Survey 2016, carried out by the National Bowel Cancer Audit [19] were used to identify whether the initial colorectal resection was carried out within an MDT with an on‐site specialist thoracic team. Twenty‐five specialist thoracic surgery centres were identified in the data out of 146 Trusts.

Multi‐level logistic regression was used to determine factors associated with the use of resection for lung metastases. Models were constructed with patients clustered within hospitals, which were then further clustered within Cancer Alliances. Explanatory variables in the risk‐adjusted model were age at resection, sex, IMD quintile, tumour site, year of primary major colorectal resection, CCI, stage at diagnosis, and whether the trust was a thoracic surgery centre. Funnel plots were constructed to show the variation in resection of metastases across MDTs using the Spiegelhalter approach [20] and those MDTs outside the 99.8% control limits were considered ‘outliers’ in terms of their practice.

Survival was calculated from the date of lung metastasectomy until death, or censored on 31 December 2016. Overall survival following pulmonary metastasectomy was analysed using Kaplan–Meier actuarial methods by sex, age at diagnosis, IMD quintile, site and stage of primary tumour at diagnosis and CCI. Log rank tests were used to test for any statistically significant difference between these groups. Cox proportional hazards regression analysis was used to determine factors associated with a higher risk of death following pulmonary metastasectomy surgery. Analyses were conducted using STATA 15 (StataCorp).

The best estimate of the representative median interval between primary colorectal cancer resection and pulmonary metastasectomy operations is 29 months, based on data from seven reports from 2006 to 2013 including a total of 1606 operations [21, 22, 23, 24, 25, 26, 27]. Our 3‐year cut‐off will have missed some cases so a sensitivity analysis was also carried out to identify pulmonary metastasectomies within 5 years of primary colorectal resection. To ensure that all patients had full 5‐year follow‐up in the data (allowing analysis of temporal trends), only patients undergoing primary resections between 2005 and 2011 were selected for sensitivity analysis. Searching for pulmonary metastasectomies within 5 years captured an additional 850 patients over the period (an average of 121 more patients annually) compared to those within 3 years. This increase in cases was balanced against the loss of 2 years’ worth of data due to requiring 5 full years of follow‐up, so all subsequent data refer to pulmonary metastasectomies taking place within 3 years of the colorectal primary resection.

## RESULTS

### Surgical management of metastases

During the period 1 January 2005 to 31 December 2013, 173 354 patients were identified as undergoing major resection for a colorectal tumour, the characteristics of whom are outlined in Table 1. Of these 3434 (2.0%) underwent one or more resections for lung metastases within 3 years of their primary colorectal resection.

**TABLE 1:** Characteristics of the study population

	No procedure (*n* = 169 920)	Pulmonary metastasectomy (*n* = 3434)	Total (*n* = 173 354)
Tumour site			
Right colon	60 043 (35.3)	727 (21.2)	60 770 (35.1)
Caecum	27 259 (16.0)	345 (10.0)	27 604 (15.9)
Appendix	862 (0.5)	6 (0.2)	868 (0.5)
Ascending colon	16 609 (9.8)	240 (7.0)	16 849 (9.7)
Hepatic flexure	5452 (3.2)	61 (1.8)	5513 (3.2)
Transverse colon	9861 (5.8)	75 (2.2)	9936 (5.7)
Left colon	60 540 (35.6)	1210 (35.2)	61 750 (35.6)
Splenic flexure	4169 (2.5)	59 (1.7)	4228 (2.4)
Descending colon	5254 (3.1)	83 (2.4)	5337 (3.1)
Sigmoid colon	38 866 (22.9)	775 (22.6)	39 641 (22.9)
Rectosigmoid	12 251 (7.2)	293 (8.5)	12 544 (7.2)
Overlapping/unspecified lesion of colon	6270 (3.7)	88 (2.6)	6358 (3.7)
Rectum	43 067 (25.3)	1409 (41.0)	44 476 (25.7)
Sex			
Male	95 279 (56.1)	2089 (60.8)	97 368 (56.2)
Female	74 641 (43.9)	1345 (39.2)	75 986 (43.8)
Age at primary colorectal resection			
≤60	33 362 (19.6)	964 (28.1)	34 326 (19.8)
61–70	48 772 (28.7)	1320 (38.4)	50 092 (28.9)
71–80	57 268 (33.7)	1029 (30.0)	58 297 (33.6)
>80	30 518 (18.0)	121 (3.5)	30 639 (17.7)
Year of primary colorectal resection			
2005	16 233 (9.6)	203 (5.9)	16 436 (9.5)
2006	17 864 (10.5)	283 (8.2)	18 147 (10.5)
2007	18 335 (10.8)	329 (9.6)	18 664 (10.8)
2008	19 103 (11.2)	390 (11.4)	19 493 (11.2)
2009	19 355 (11.4)	390 (11.4)	19 745 (11.4)
2010	19 975 (11.8)	452 (11.8)	20 427 (11.8)
2011	20 092 (11.8)	461 (13.4)	20 553 (11.9)
2012	20 195 (11.9)	492 (14.3)	20 687 (11.9)
2013	18 768 (11.0)	434 (12.6)	19 202 (11.1)
Tumour stage at diagnosis			
I	23 353 (13.7)	326 (9.5)	23 679 (13.7)
II	58 088 (34.2)	988 (28.8)	59 076 (34.1)
III	57 394 (33.8)	1266 (36.9)	58 660 (33.8)
IV	13 322 (7.8)	511 (14.9)	13 833 (8.0)
Unknown	17 763 (10.5)	343 (10.0)	18 106 (10.4)
IMD quintile			
1, least deprived	36 582 (21.5)	739 (21.5)	37 321 (21.5)
2	38 637 (22.7)	832 (24.2)	39 469 (22.8)
3	35 746 (21.0)	687 (20.0)	36 433 (21.0)
4	31 661 (18.6)	590 (17.2)	32 251 (18.6)
5, most deprived	27 294 (16.1)	586 (17.1)	27 880 (16.1)
Charlson Comorbidity Index			
0	135 383 (79.7)	2881 (83.9)	138 264 (79.8)
1	23 878 (14.1)	434 (12.6)	24 312 (14.0)
2	6705 (3.9)	90 (2.6)	6795 (3.9)
≥3	3954 (2.3)	29 (0.8)	3983 (2.3)

Note:

Values in parentheses are percentages.

Abbreviation: IMD, index of multiple deprivation.

There were 2941 patients (85.6%) having one surgical episode, 431 (12.6%) having two episodes and 62 (1.8%) having three or more within 3 years, giving a total of 3998 pulmonary metastasectomy episodes. Looking at the extent of the first pulmonary procedure, 25 (0.7%) were pneumonectomies, 23 (0.7%) were bilobectomies, 895 (26.1%) were lobectomies and 2491 (72.5%) were sublobar resections. There were 574 patients (16.7%) who had previously had a liver resection at the time of their pulmonary resection, 85 (2.5%) who had a subsequent liver resection and seven patients (0.2%) had liver and pulmonary metastasectomy on the same day.

**FIGURE 1:**
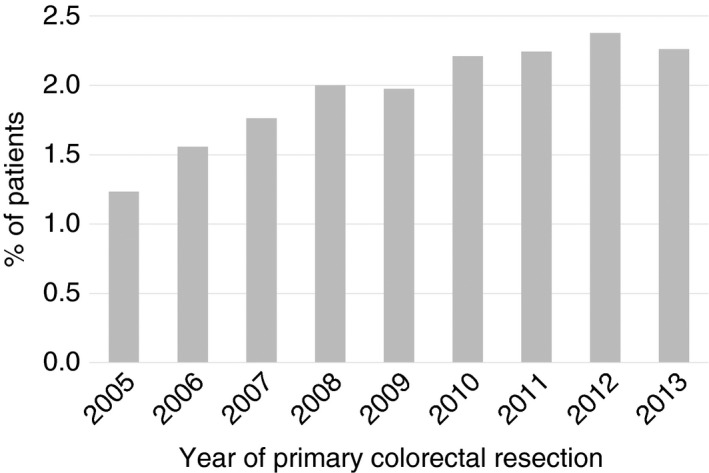
Percentage of patients receiving a pulmonary resection within 3 years of their primary colorectal resection as a percentage of all patients undergoing major colorectal resection

The percentage of patients receiving pulmonary metastasectomy increased from around 1.2% of all patients in 2005, who underwent a major resection for colorectal cancer, to 2.3% in 2013 (Figure 1). The rate of pulmonary metastasectomy was higher for patients presenting with tumours in the rectum (3.17%) than for tumours of the colon (1.49%) which is a reflection of the well documented difference in incidence due to the anatomical difference in venous drainage between the colon and rectum [2, 28, 29].

Multi‐level logistic regression was performed using data from 2010 to 2013, during which period staging of the primary tumour was more complete (Table 2). The likelihood of receiving surgery reduced with age (OR 0.23 for age >80 compared to the average age group 61–70; 95% CI 0.18–0.29). There was no significant difference in the rate of surgery between men and women or across deprivation quintiles. The odds of resection decreased with increasing CCI (OR 0.44 for CCI 3 or more compared to a score of zero; 95% CI 0.27–0.71). Patients with rectal tumours were most likely to receive surgery (OR 2.61; 95% CI 2.30–2.97) and those with tumours in the right colon were the least likely to receive treatment for lung metastases. Patients with more advanced disease stage at diagnosis were more likely to undergo pulmonary metastasectomy within 3 years of their primary colorectal resection (OR for Stage IV vs. Stage III 1.77; 95% CI 1.55–2.03). These will include patients with synchronous metastases, which were already evident at the time of initial cancer staging. There was no statistically significant difference in the likelihood of pulmonary metastasectomy depending on whether patients received surgery for their colorectal primary in a trust with a specialist thoracic centre, in either the risk‐adjusted or non‐adjusted model.

**TABLE 2:** Odds of having a pulmonary metastasectomy within 3 years of primary colorectal tumour resection

	Unadjusted odds ratio	95% confidence interval	95% confidence interval	*P*	P across groups	Adjusted odds ratio	95% confidence interval	95% confidence interval	P	P across groups
Year of resection of colorectal primary	1.01	0.97	1.06	0.541	0.541	1.01	0.96	1.05	0.812	0.812
Primary resection carried out in hospital with thoracic centre	1.07	0.96	1.20	0.236	0.236	1.05	0.90	1.23	0.519	0.519
Age at resection of colorectal primary										
≤60	1.08	0.96	1.22	0.182	<0.001	1.02	0.91	1.15	0.741	<0.001
61–70	1.00					1.00				
71–80	0.73	0.66	0.82	<0.001		0.81	0.71	0.92	0.001	
>80	0.19	0.15	0.24	<0.001		0.24	0.19	0.30	<0.001	
Sex										
Male	1.00				0.001	1.00				0.598
Female	0.85	0.77	0.93	0.001		0.97	0.89	1.07	0.598	
IMD quintile										
1, least deprived	1.00				0.209	1.00				0.299
2	1.07	0.94	1.23	0.304		1.08	0.94	1.24	0.293	
3	0.98	0.85	1.14	0.829		0.99	0.85	1.14	0.872	
4	0.93	0.80	1.08	0.315		0.92	0.79	1.07	0.281	
5, most deprived	1.09	0.94	1.27	0.266		1.04	0.89	1.22	0.613	
Stage of primary tumour at diagnosis										
I	0.57	0.48	0.67	<0.001	<0.001	0.50	0.42	0.59	<0.001	<0.001
II	0.76	0.68	0.85	<0.001		0.87	0.78	0.98	0.024	
III	1.00					1.00				
IV	1.65	1.44	1.89	<0.001		1.77	1.55	2.03	<0.001	
Unknown	0.83	0.69	1.01	0.061		0.72	0.59	0.87	0.001	
Tumour site										
Right colon	1.00				<0.001	1.00				<0.001
Left colon	1.63	1.43	1.85	<0.001		1.52	1.34	1.73	<0.001	
Rectum	2.73	2.42	3.09	<0.001		2.61	2.30	2.96	<0.001	
Colon unknown	1.53	1.12	2.10	0.008		1.50	1.09	2.06	0.013	
Charlson Comorbidity Index										
0	1.00				<0.001	1.00				<0.001
1	0.91	0.80	1.03	0.129		1.03	0.90	1.18	0.644	
2	0.56	0.42	0.74	<0.001		0.72	0.54	0.96	0.024	
≥3	0.30	0.19	0.49	<0.001		0.44	0.27	0.71	0.001	

Abbreviation: IMD, index of multiple deprivation.

**FIGURE 2:**
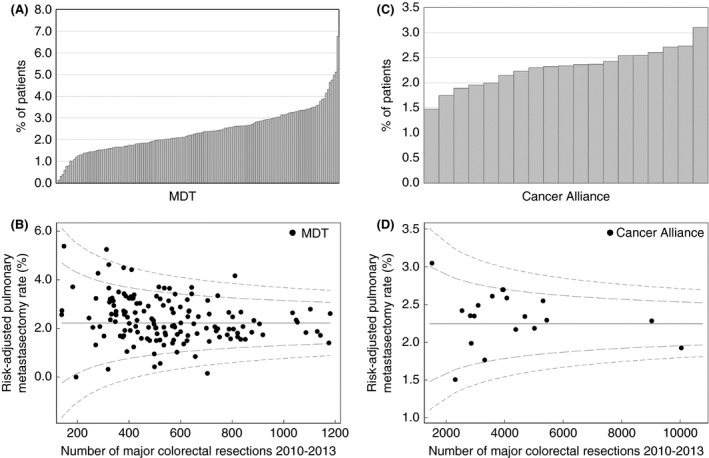
Organization funnel plots showing the variation in (A) unadjusted pulmonary metastasectomy rate by trust, (B) risk‐adjusted pulmonary metastasectomy rate by trust, (C) unadjusted pulmonary metastasectomy rate by Cancer Alliance and (D) risk‐adjusted pulmonary metastasectomy rate by Cancer Alliance

There was a large degree of variation in the proportion of patients having a pulmonary metastasectomy when analysed by the hospital provider for their primary colorectal resection, with crude and risk‐adjusted rates both between 0.0% and 6.8% (*P* < 0.001). By Cancer Alliance/Vanguard the crude rate varied between 1.5% and 3.1% (*P* = 0.003) and the risk‐adjusted rate varied between 1.5% for Lancashire and Cumbria and 3.0% for South East London; however, there was no statistically significant difference between Cancer Alliances following risk adjustment (Figure 2).

### Survival

**FIGURE 3:**
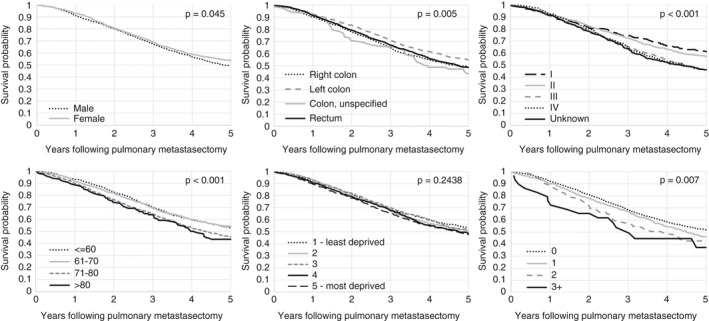
Kaplan–Meier survival curves grouped by sex, tumour site, tumour stage, age and diagnosis, IMD quintile and Charlson Comorbidity Index score

Overall median survival following pulmonary metastasectomy was 44 months, with 1, 3 and 5 years at 91.6%, 67.9% and 50.8% respectively. Thirty‐day and 90‐day postoperative mortality were 0.6% and 1.2% respectively. Women had higher overall survival than men (5‐year survival of 53.6% vs. 49.0%, *P* = 0.045); however, once adjusted for patient and tumour characteristics, this difference was not significant. Older patients had reduced survival following lung metastasectomy (5‐year survival of 53.0% for those ≤60 compared to 43.5% for those >80, adjusted HR 1.68, 95% CI 1.30–2.16) (Table 3). There was no statistically significant difference in overall survival across deprivation quintile (5‐year survival of 53.8% for least deprived vs. 47.8% for most deprived); however, when adjusted for patient and tumour characteristics there was a statistically significant reduction in survival after pulmonary metastasectomy for the most deprived group compared to the least deprived group (HR 1.18, 95% CI 1.02–1.37, *P* = 0.030). There was an increase in HR with increasing comorbidity (HR 1.78, 95% CI 1.13–2.81). Patients with primary tumours in the left colon had improved survival compared to all other sites (HR 0.87, 95% CI 0.76–0.99; Figure 3).

**FIGURE 4:**
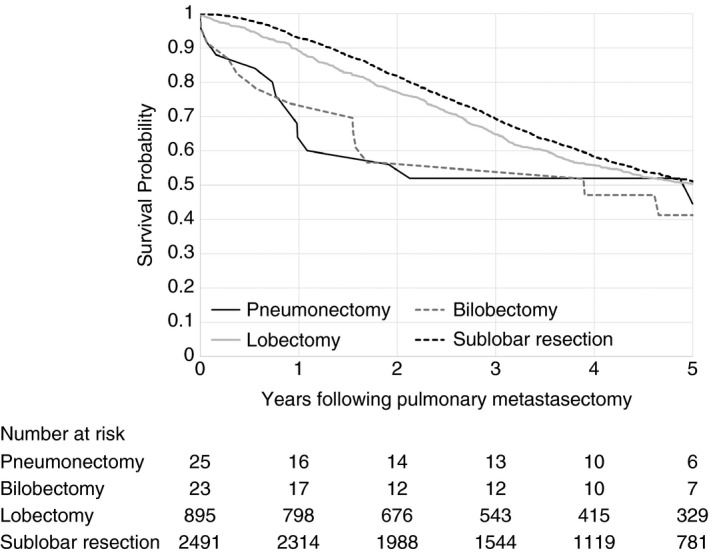
Kaplan–Meier survival curves grouped by extent of pulmonary metastasectomy surgery

Patients who had a prior liver resection had a 5‐year survival of 37.8% post‐pulmonary metastasectomy, whilst those who had not undergone a liver resection had a 5‐year survival of 53.7% following pulmonary metastasectomy (*P* < 0.001). The Kaplan–Meier survival curve in Figure 4 suggests improved survival for those undergoing sublobar excision compared to lobectomy until around 5 years, when there is no observed difference between the two procedures. It is not possible to know how much this association was influenced by the severity of the disease or the treatment. There are few pneumonectomies and bilobectomies and survival was much worse for these patients compared to those undergoing lobectomy; again the disease and the treatment was more severe.

**TABLE 3:** Cox proportional hazards model results for survival following pulmonary metastasectomy and survival following primary colorectal resection

	Pulmonary metastasectomy							
	Unadjusted HR	95% CI		*P*	Adjusted HR	95% CI		*P*
Year of resection of colorectal primary	0.96	0.94	0.98	<0.001	0.95	0.93	0.97	<0.001
Primary colorectal resection carried out in hospital with thoracic centre	1.07	0.96	1.20	<0.203	1.05	0.94	1.17	0.413
Age at resection of colorectal primary								
≤60	1.00				1.00			
61–70	1.00	0.88	1.12	0.944	1.02	0.90	1.15	0.727
71–80	1.30	1.15	1.47	<0.001	1.38	1.22	1.56	<0.001
>80	1.48	1.16	1.89	0.002	1.68	1.30	2.16	<0.001
Sex								
Male	1.00				1.00			
Female	0.86	0.78	0.94	0.002	0.86	0.78	0.95	0.003
IMD quintile								
1, least deprived	1.00				1.00			
2	1.05	0.92	1.21	0.469	1.05	0.91	1.20	0.533
3	1.06	0.92	1.23	0.435	1.05	0.90	1.21	0.541
4	1.14	0.98	1.32	0.100	1.15	0.99	1.34	0.074
5, most deprived	1.17	1.01	1.36	0.036	1.18	1.02	1.37	0.030
Stage of primary tumour at diagnosis								
I	1.00				1.00			
II	1.15	0.95	1.40	0.143	1.14	0.94	1.39	0.183
III	1.54	1.28	1.85	<0.001	1.60	1.33	1.93	<0.001
IV	1.58	1.29	1.94	<0.001	1.74	1.41	2.14	<0.001
Unknown	1.55	1.25	1.93	<0.001	1.53	1.23	1.91	<0.001
Tumour site								
Right colon	1.00				1.00			
Left colon	0.85	0.74	0.96	0.010	0.86	0.76	0.98	0.022
Rectum	0.98	0.89	1.11	0.740	1.01	0.89	1.14	0.910
Colon unknown	1.18	0.87	1.57	0.258	1.18	0.88	1.57	0.266
Charlson Comorbidity Index								
0	1.00				1.00			
1	1.13	0.98	1.30	0.093	1.14	0.99	1.31	0.068
2	1.37	1.04	1.81	0.026	1.36	1.03	1.80	0.031
≥3	1.80	1.14	2.82	0.011	1.78	1.13	2.81	0.013

Abbreviations: HR, hazard ratio; IMD, index of multiple deprivation.

Potential volume effects on survival outcomes were considered; however, there was no statistically significant difference in 5‐year survival following pulmonary metastasectomy between high, mid and low volume providers. There was no correlation between the crude or adjusted pulmonary resection rate and 5‐year survival by Cancer Alliance or MDT.

## DISCUSSION

This large population‐based study demonstrates that there has been an increase in the rate of pulmonary metastasectomy between 2005 and 2013, with the rate remaining constant at around 2.3% for the later years of 2010–2013. Increases in the frequency of pulmonary metastasectomy were also observed in the American National Inpatient Sample between 2000 and 2011 [30]. Previous studies have estimated the incidence of pulmonary metastasectomy to be within 1%–2.5% for all colorectal cancer patients [14, 16]. The English NHS national figure of 2.3% is at the higher end of this range. When considering the number of patients receiving pulmonary metastasectomy, within 3 years of diagnosis, as a proportion of all colorectal cancer patients diagnosed with their first tumours between 2005 and 2013, then a figure of 1.36% for the English NHS over the whole series and 1.5% for the later years 2010–2013 is obtained. There appear to be no large population‐based studies to enable direct comparison. The rate of pulmonary metastasectomy in patients with rectal cancer was twice that for patients with tumours in the colon. This reflects the higher rate of metastasis to the lungs in rectal cancer, related to the anatomical differences in venous drainage already noted [2, 28, 29].

Postoperative 30‐ and 90‐day mortality of 0.6% and 1.2% respectively shows lung metastasectomy to be relatively safe. Overall 5‐year survival following pulmonary metastasectomy was 50.8%. This national figure falls within the range reported by a number of single‐centre studies which report rates varying between 27% and 66% [8, 31, 32].

Receiving surgery for the primary colorectal tumour, in a trust with a specialist thoracic surgical centre on‐site, did not increase the chances of a patient receiving pulmonary resection in either the risk‐adjusted or unadjusted model. This contrasts with liver metastasectomy whereby patients with colorectal cancer liver metastases are more likely to receive surgical resection when their primary colorectal tumour is resected in a hospital with an on‐site hepatobiliary team [33]. This also contrasts with studies carried out on primary non‐small‐cell lung cancer, which suggest that being seen first in a specialist thoracic surgical centre increases the likelihood of receiving lung surgery [34].

There was significant variation in the percentage of patients receiving a pulmonary resection between trusts, with two trusts having higher than average pulmonary metastasectomy rates, that is, outside of the 99.8% confidence limits so outside of the ‘random’ variation that would be expected, and one trust having a lower than average pulmonary metastasectomy rate of only 0.14% of patients. Whilst there were significant differences in the crude rate of pulmonary metastasectomy by the Cancer Alliance within which the patient received their colorectal resection, this was no longer observed following risk adjustment for patient and tumour characteristics. Therefore it seems that referral practice is similar at a regional level, with more variation occurring on a hospital site level. This variation may also be affected by trusts referring patients for ablation or SBRT rather than resection. Data on ablation were not fully captured during this work due to a large amount of variation in coding practice. Exploring this will form the basis for future work. Current NICE draft guidance [7] suggests that there is insufficient evidence to recommend one type of local treatment over another so either surgical resection, ablation or SBRT should be considered for people with colorectal lung metastases. It will be interesting to note whether trusts with lower resection rates refer larger numbers of patients for ablation or SBRT and whether patients have similar access to all treatments across trusts.

The rate of pulmonary metastasectomy in the over 80s group was almost one‐fifth of that for patients aged 71–80; however, there was no statistically significant difference in 5‐year survival between the two age groups. Five‐year survival following surgery for lung metastases for the over 80s group was 43.5%. The postoperative mortality and 5‐year survival outcomes in the group receiving metastasectomy, along with the small numbers of over 80s undergoing lung surgery, suggests that the threshold for lung metastasectomy is extremely high within this population and only the fittest patients receive surgery. However, older patients may be more likely to undergo less invasive ablative treatments rather than resection. Unfortunately, we do not have information on the frequency of ablative treatments or radiotherapy in order to assess whether they receive alternative treatment.

There was no statistically significant difference in the likelihood of receiving a pulmonary metastasectomy across different deprivation quintiles, in contrast to the trend observed for resection for colorectal cancer liver metastases [35] or resection for primary lung cancer where more deprived patients are less likely to receive pulmonary resection [36, 37].

One limitation of this study is that there is not sufficient information to ascertain the reason for the lung resection. It has been presumed that these surgical procedures are taking place due to pulmonary metastases, given the proximity to diagnosis of colorectal cancer; however, it is also possible that patients received surgery for other diagnoses such as primary lung cancer or emphysema.

## CONCLUSION

This study comprises the first large population‐based analysis of the use of pulmonary metastasectomy for colorectal cancer lung metastases. The rate of pulmonary metastasectomy in the population undergoing major colorectal resection within the NHS is around 2.3% in the later part of the study; however, it must be noted that this figure does not provide a full picture of the number of patients undergoing local treatment for pulmonary metastasis, as it does not account for those who may be undergoing IGTA or SABR as an alternative treatment to surgery. Previous studies have shown that the likelihood of receiving liver resection surgery for metastatic colorectal cancer is affected by the presence of an on‐site specialist hepatobiliary centre in the hospital where the patient first receives their colorectal resection. In this study, there was no statistically significant difference in the likelihood of pulmonary metastasectomy depending on whether the patient underwent their colorectal surgery in a hospital with a specialist thoracic centre on‐site or not. There was variation in the rate of pulmonary metastasectomy across hospitals, however, which remained after accounting for patient and tumour characteristics. Further randomized studies are urgently required to establish if there is a survival benefit from local treatment of lung metastases and, if so, for which patients. Such studies should allow optimization of the management of pulmonary metastases from colorectal cancer in the English NHS.

## CONFLICT OF INTERESTS

None.

## ETHICAL APPROVAL

This work involves patient‐level information collected by the NHS that has either been provided by, or derived from, patients as part of their care and support. The data are collated, maintained and quality assured by the National Cancer Registration and Analysis Service, which is part of Public Health England (PHE). Access to the data was facilitated by the PHE Office for Data Release. The data used for this study are available from the National Cancer Registration and Analysis Service via the PHE Office for Data Release, subject to relevant approvals. This work was supported by the Cancer Research UK (grant number A23706) and Yorkshire Cancer Research (grant number L394). It was underpinned by the Leeds MRC Medical Bioinformatics Centre (grant number MR/L01629X/1). This study was approved by the South West & Central Bristol Research Ethics Committee (ref 18/ SW/0134).

## AUTHOR CONTRIBUTIONS

Hayley M Fenton analysed the data and wrote the draft manuscript with input from all authors. John C Taylor oversaw statistical analysis. Eva J Morris (Principal Investigator) and Tom Treasure conceived the study and methods.

## Supporting information

Table S1

## Data Availability

Data are available through CORECT‐R and PHE subject to relevant approvals. Due to the nature of the data used they are not available directly from the authors.
