# Supplementary material for: National variation in pulmonary metastasectomy for colorectal cancer
Source: Colorectal Dis. 2021 Jan 24;23(6):1306–16. doi: 10.1111/codi.15506 (PMC8614123; doi:10.1111/codi.15506)
Supplement: Supplementary file 1 — Table S1 [file CODI-23-1306-s001.docx]

**APPENDIX**

Supplementary Table 1: OPCS 4.8 codes for major colorectal resection and pulmonary metastasectomy

| **OPCS code** | **Description** |
| --- | --- |
| Primary colorectal resection |  |
| H04 | Total excision of colon and rectum |
| H05 | Total excision of colon |
| H06 | Extended excision of right hemicolon |
| H07 | Other excision of right hemicolon |
| H08 | Excision of transverse colon |
| H09 | Excision of left hemicolon |
| H10 | Excision of sigmoid colon |
| H11 | Other excision of colon |
| H29 | Subtotal excision of colon |
| H33 | Excision of rectum |
| X14 | Clearance of pelvis |
| Pulmonary metastasectomy |  |
| E541 | Total pneumonectomy |
| E542 | Bilobectomy of lung |
| E543 | Lobectomy of lung |
| E544 | Excision of segment of lung |
| E545 | Partial lobectomy of lung NEC |
| E548 | Other specified excision of lung |
| E549 | Unspecified excision of lung |
| E552 | Open excision of lesion of lung |
| E553 | Open cauterisation of lesion of lung |
| E554 | Open destruction of lesion of lung NEC |
| E558 | Other specified open extirpation of lesion of lung |
| E559 | Unspecified open extirpation of lesion of lung |
| E574 | Incision of lung NEC |
| E578 | Other specified other open operations on lung |
| E598 | Other specified other operations on lung |
